# PDE5 Overexpression in Well-Differentiated Thyroid Carcinomas Is Associated with Lymph Node Metastasis

**DOI:** 10.1155/2017/6243932

**Published:** 2017-10-01

**Authors:** Ning Zhang, Zeng Fang, Qiufang Li, Kebing Wang, Songqi Li, Wen Li, Shenming Wang

**Affiliations:** ^1^Division of Breast and Thyroid Surgery, First Affiliated Hospital, Sun Yat-sen University, Guangzhou, China; ^2^Laboratory of General Surgery, First Affiliated Hospital, Sun Yat-sen University, Guangzhou, China; ^3^Division of Vascular Surgery, First Affiliated Hospital, Sun Yat-sen University, Guangzhou, China

## Abstract

Overexpression of PDE5 is observed in certain human cancers, but PDE5 expression in well-differentiated thyroid carcinoma (WDTC) is unknown. We therefore examined PDE5 expression and its relationship with the clinicopathological features of WDTC. Real-time qPCR and Western blotting were performed to analyze the expression of PDE5 mRNA and protein in paired WDTC tumor and adjacent nontumor tissues. Immunohistochemistry was used to analyze the expression of PDE5 in paraffin-embedded tissues obtained from 103 cases of WDTC. Statistical analyses were performed to examine the correlation between PDE5 expression and clinicopathological features. The expression of PDE5 mRNA and protein was upregulated in WDTC lesions compared to their paired noncancerous tissues. The expression of PDE5 was significantly correlated with age (*P* = 0.032), regional lymph node status (*P* = 0.004), and the presence of distant metastasis (*P* = 0.020). High PDE5 expression was more closely associated with lymph node involvement in patients over 45 years (OR = 15.60, *P* ≤ 0.05). Thus, PDE5 may be a potential biomarker in WDTC, particularly in patients with regional lymph node metastasis, which is associated with disease recurrence, treatment failure, and morbidity. PDE5 expression may also help predict the prognosis and recurrence of WDTC after surgery.

## 1. Introduction

Thyroid cancer (TC) is the most common endocrine malignancy; the incidence of which has been steadily increasing over the last three decades [1]. Papillary and follicular TCs are the most common forms of TC, and they are classified as well-differentiated thyroid cancer (WDTC) based on the histological features. WDTCs are generally indolent tumors that are associated with low mortality. Although the 30-year disease-specific survival rates can exceed 95%, the 5-year survival is as low as 56% in patients with metastatic disease [2]. Disease recurrence or persistence is associated with high mortality and long-term treatment failure [1–4]. Regional lymph node involvement is often observed in the early stage of the disease and is associated with postoperative recurrence [5–8]. Therefore, accurate evaluation of regional lymph node status plays an important role in WDTC treatment. Fine-needle aspiration biopsy (FNAB) is a critical diagnostic test for evaluation of the nodules. However, the procedure is invasive and is associated with a misdiagnosis rate of 10–15%; this limits the application of FNAB in clinical practice [3]. Therefore, it would be useful to identify other methods for evaluating the regional lymph node status in WDTC.

PDE5 is a cytoplasmic enzyme that regulates the levels of cGMP by hydrolyzing it [9]. It plays a critical role in cell growth and apoptosis [10]. Previous studies have shown that PDE5 is overexpressed in human prostate cancer, nonsmall cell lung cancer, and breast cancer [11–14]. PDE5 inhibitors have also been used for therapeutic treatment [10]. Passon and colleagues have reported that the copy number of PDE5 is amplified in papillary thyroid cancer (PTC) [15]. Further, it was reported that variation in the copy number of PDE5 contributes to tumorigenesis and the development of PTC [16]. Sodium iodide symporter (NIS) is associated with the differentiation of TC and postoperative ^131^I treatment resistance [17]. Sponziello et al. found that PTC tissues with high levels of PDE5 mRNA had a lower level of NIS transcripts, which means that poor differentiation might lead to long-term treatment failure [18]. Thus, PDE5 overexpression may play a role in the cell growth and metastasis of PTC. However, the significance of PDE5 in WDTC patients is unknown. Therefore, in the present study, we examined PDE5 expression in 103 formalin-fixed, paraffin-embedded tissue specimens of human WDTC and its correlation with the clinicopathologic features of the patients.

## 2. Materials and Methods

### 2.1. Patients and Samples

Paraffin-embedded samples were obtained from 103 patients diagnosed with WDTC who underwent surgery between July 2009 and December 2015 at the Department of Breast and Thyroid Surgery and Department of Pathology, the First Affiliated Hospital of Sun Yat-sen University. All the patients' ages ranged from 7 to 81 years (median = 50). The patient group included 19 and 57 patients who were diagnosed with WDTC with distant metastasis and lymph node metastasis, respectively, confirmed by imaging or pathological examination, and 27 patients with primary WDTC. Clinicopathological information such as age, tumor size, lymph node status, and histological type was obtained by reviewing their medical records and pathology reports.

Four matched pairs of fresh WDTC tumor and adjacent noncancerous tissue samples (at least 2 cm away from the margin of the tumor tissue) were also obtained for determining the mRNA and protein levels of PDE5. Histopathological analysis with hematoxylin-eosin staining of frozen sections confirmed that the tumor tissues comprised >70% cancer cells without necrosis and that no cancer lesions were present in the noncancerous tissues.

The study was approved by the Medical Ethical Committee of the First Affiliated Hospital of Sun Yat-sen University (Guangzhou, China). Informed consent was obtained from all the patients for the use of their clinical specimens.

### 2.2. RNA Extraction and Real-Time qPCR

We followed the methods of Zhang et al. [19]. Total RNA from the primary tumor and adjacent nontumor tissue samples was extracted using TRIzol reagent (Invitrogen, Carlsbad, CA, USA) according to the manufacturer's instructions. RNA concentration and quality were assessed spectrophotometrically at 260 and 280 nm. The enzyme and reagents for reverse transcription and PCR amplification were obtained from Roche. The cDNAs were amplified by PCR (42°C for 15 min, 85°C for 5 min, and 65°C for 15 min) using a thermal cycler (Hema 9600, Applied Biosystems). Real-time qPCR was performed using a Roche LC480 sequence detection system (Roche, Swiss). The following published primer sequences were used for the reactions [20]: PDE5, sense (5′- AAGCAAATGGTCACATTGGA -3′) and antisense (5′- TCTGGAAGTTCTGCACAAGG -3′); GAPDH, sense (5′-CTGACTTCAACAGCGACACC-3′) and antisense (5′-TGCTGTAGCCAAATTCGTTG-3′). The PCR protocol used was as follows: denaturation at 95°C for 30 s, followed by 40 cycles of annealing for 20 s each at 60°C. Expression data were normalized to the geometric mean of a GAPDH housekeeping gene.

### 2.3. Western Blot Analysis

Four matched pairs of WDTC tumor tissues and adjacent nontumor tissues were harvested and lysed in 50 mM Tris (pH 7.5), 100 mM NaCl, 1 mM EDTA, 0.5% NP40, 0.5% Triton X-100, 2.5 mM sodium orthovanadate, 10 *μ*M of a protease inhibitor cocktail, and 1 mM phenylmethylsulfonyl fluoride. Equal amounts of protein were electrophoretically separated on a 9% SDS polyacrylamide gel and transferred to polyvinylidene fluoride membranes (Millipore, Bedford, MA, USA). The membranes were incubated at 4°C overnight with anti-human PDE5 rabbit monoclonal antibody (ab64179, 1 : 1000; Abcam Inc., Cambridge, USA). PDE5 expression was detected with horseradish peroxidase- (HRP-) conjugated goat anti-rabbit IgG secondary antibody (1 : 10,000; Cell Signaling Technology, USA). The immunoreactive bands were visualized with an ECL chemiluminescence system (Tanon, Beijing, China). Anti-*β*-actin mouse monoclonal antibody (1 : 1000; Cell Signaling Technology, USA) was used as a loading control.

### 2.4. Immunohistochemistry

Immunohistochemical staining was carried out on formalin-fixed, paraffin-embedded sections (4 *μ*m thick) that were deparaffinized in xylene and rehydrated in an ethanol series with decreasing concentrations and rinsed in phosphate-buffered saline. Following this, antigen retrieval was performed with microwave treatment in 10 mM citrate buffer (pH 6.0). Immunohistochemical staining was carried out using the EnVision™ Kit (Dako, Hamburg, Denmark) according to the manufacturer's instructions. Endogenous peroxidase activity was quenched by treatment with 3% hydrogen peroxide for 15 min. The sections were then incubated with primary anti-rabbit antibody against PDE5 (ab64179, 1 : 500) overnight at 4°C. Then, the tissue sections were sequentially incubated with ready-to-use HRP immunoglobulin (EnVision Kit) for 30 min and developed with 3,3′-diaminobenzidine as a chromogen substrate. The nuclei were counterstained with Meyer's hematoxylin.

The extent of PDE5 immunostaining was evaluated independently by two pathologists who were blinded to the survival outcomes of the participants. They determined the proportion of positively stained tumor cells (staining area) and the intensity of staining. Staining intensity was scored as follows: 0, no staining; 1, weak staining (light yellow color); 2, moderate staining (yellow brown color); and 3, strong staining (brown color). The proportion of positively stained tumor cells was scored as follows: 0, no positively stained tumor cells; 1, <5% positively stained tumor cells; 2, 6–25% positively stained tumor cells; 3, 26–50% positively stained tumor cells; and 4, >50% positively stained tumor cells. A modified immunoreactivity scoring method was used to evaluate the immunostaining results by multiplying staining intensity with the staining area (staining index, SI) as previously described [20]. The PDE5 expression level in breast carcinoma lesions was determined by the SI, which was assigned values of 0, 1, 2, 3, 4, 6, 9, or 12. The optimal cutoff value was identified as follows: An SI score of >6 was considered to indicate high PDE5 expression, while an SI score of ≤6 was considered to indicate low PDE5 expression.

### 2.5. Statistical Analysis

Chi-square test and logistic regression analysis were used for analysis of differences between the groups. Differences were considered significant when the *P* value was ≤0.05. SPSS v.23 was used for statistical analysis.

All cases were risk stratified on the basis of the clinical and pathological features in accordance with the 2015 American Thyroid Association Risk Stratification System Guidelines, TNM staging, GAMES, and Mayo Clinic Scoring System (metastases, age, complete resection, invasion, size, or MACIS).

## 3. Results

### 3.1. Upregulation of PDE5 Expression in WDTC

**Figure 1:**
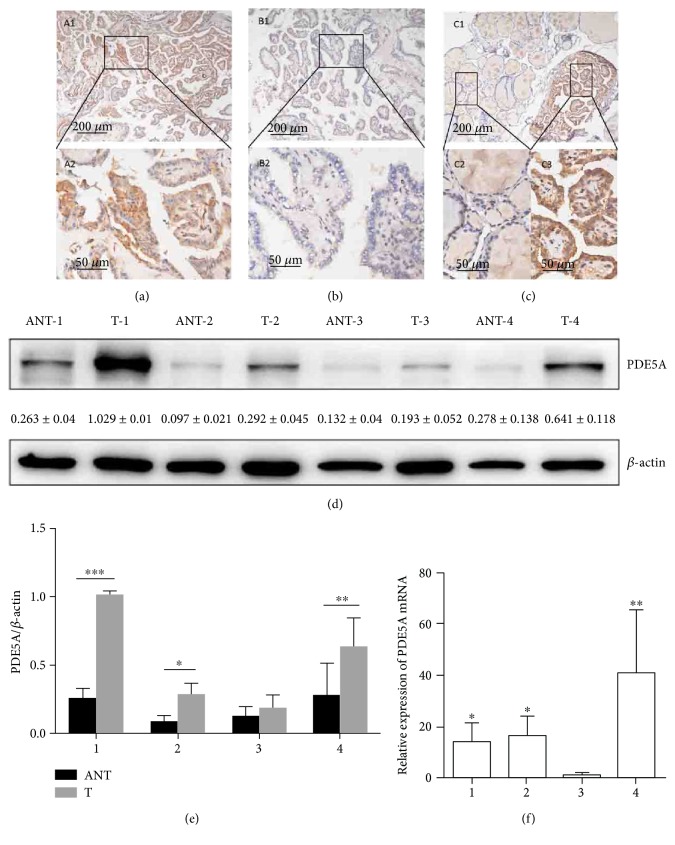
Upregulation of PDE5 expression in well-differentiated thyroid carcinoma (WDTC). (a, b, c) Expression of PDE5 in WDTCs as determined by immunohistochemistry. Strong staining (A1, ×10; A2, ×40) and negative staining (B1, ×10; B2, ×40) can be observed. A WDTC sample (C1, ×10) with strong PDE5 staining in the carcinoma tissues (C2, ×40) and normal thyroid follicular epithelial cells with weak staining (C3, ×40). (d and e) Western blots show the expression levels of the PDE5 protein in four WDTC tumor tissues (T) and their paired adjacent noncancerous tissues (ANT); *β*-Actin was used as the loading control. (f) Real-time qPCR analysis results show the expression levels of PDE5 mRNA in WDTC tumor tissues relative to their paired adjacent noncancerous tissues (T/ANT). Values are given as the ratio of PDE5 expression to GAPDH expression (^∗^*P* < 0.05, ^∗∗^*P* < 0.01, and ^∗∗∗^*P* < 0.001).

Real-time qPCR and Western blot analysis showed that the expression levels of PDE5 mRNA and protein, respectively, were markedly higher in all four WDTC lesions than in their paired adjacent noncancerous tissues (Figures 1(d), 1(e), and 1(f)). All four cases were female, and their histological types were thyroid papillary carcinoma confirmed by pathology. The detail clinicopathological features were described in Table 1.

Furthermore, the immunohistochemical staining patterns revealed that PDE5 was mainly localized in the cytoplasm of the tumor cells. No staining or weak cytoplasmic staining was detectable in the follicular epithelial cells of the adjacent noncancerous thyroid tissue (Figure 1(c)).

### 3.2. Relationship between PDE5 Expression and the Clinicopathologic Features of WDTC

The immunohistochemical staining results showed that 52 (50.5%) of the 103 WDTC patients had a high level of PDE5 expression (SI > 6), whereas 51 (49.5%) had a low level of PDE5 expression (SI ≤ 6).

The correlation between PDE5 protein expression and the clinicopathologic features of WDTC is shown in Table 2. PDE5 expression was significantly correlated with age (*P* = 0.032), the regional lymph node status (*P* = 0.004), and the presence of distant metastasis (*P* = 0.020), according to the results of the chi-square (*χ*^2^) test. We observed more significant differences in lymph node metastasis in patients who were over 45 years old (OR = 15.60, *P* ≤ 0.05) (Table 3).

## 4. Discussion

This study found a significant relationship between PDE5 expression and lymph node involvement in WDTC. PDE5 may be a potential marker of regional lymph node involvement and disease prognosis in WDTC. The results would be valuable from the viewpoint of future diagnosis.

At variance with cAMP and its pathway, widely investigated as the main mediator of the TSH effects on thyroid cells, previous studies are widely focused on the TSH effect regulation of cAMP and its pathway on thyroid cells and involved in several thyroid diseases [21]. The role of the regulation of cGMP, mainly exerted by PDEs, in thyroid cancer is unclear. Sponziello et al. [18] found higher expression levels of PDE5A in tumors versus normal tissues in a group of 86 PTCs and noted the presence of higher expression levels of PDE in PTCs with BRAF V600E mutation. In PTCs, activating BRAF mutation represents the most frequent genetic alteration and is also considered as a marker of aggressiveness [22–25]. However, the significance of PDE5 expression with clinicopathologic features in these PTCs was unobserved. We confirmed that PDE5 expression was upregulated in WDTC tissues at both the mRNA and protein level compared to adjacent nontumor tissues. Further, our results revealed that overexpression of PDE5 in WDTC was significantly correlated with age, lymph node metastases, and distant metastases in a larger group of 103 WDTCs.

The role of PDE5 in cancer pathogenesis and prognosis is uncertain and complex. Peak et al. [26] concluded that PDE5 inhibition may help decrease the risk of developing colorectal and breast cancers, leukemia, and myeloma. PDE5 expression was found to have significantly higher levels in malignant breast tumors and had positive correlation with tumor grade, stage, and lymph node involvement [27]. Through cGMP/PKG-dependent neoplastic pathway, inhibition of PDE5 can induce apoptosis and prevent tumorigenesis in colon and breast tumor cells [28–30]. However, conflicting data suggest that inhibition of the NO/cGMP pathway can increase cellular motility, invasiveness, and cancer progression in breast tumor cells [31]. Previous study found that PDE5 inhibitors can block the proliferation of thyroid cancer cells in culture [18]. Further studies in vivo and vitro are needed to explain the mechanism of why PDE5 overexpression can be a driver of tumorigenesis and clarify whether specific inhibition of PDE5 may be proposed for the treatment of these tumors.

Cervical lymph node metastasis has been reported to be present in 20–90% of patients with WDTC [8, 16, 32–34]. The risk of regional recurrence is higher in patients with lymph node metastases, especially in those with more than 10 involved nodes and extracapsular extension [34]. The presence of lymph node metastasis is also indicative of a poorer prognosis [17]. Because regional lymph node metastasis is a significant indicator of disease prognosis or recurrence in clinical practice, it is important to determine whether regional lymph node metastasis is involved before surgery. In the present study, we observed high PDE5 expression in patients positive for regional lymph node metastases. Therefore, PDE5 may have potential for the identification of patients in whom aggressive treatment, such as prophylactic central lymph node dissection, would be beneficial. Since these results are only preliminary, tissue samples from more cases of WDTC may be required to confirm the significance of PDE5 in lymph node metastases.

## 5. Conclusions

In summary, our study investigated PDE5 expression in WDTC. We found high PDE5 expression in patients with lymph node metastasis; moreover, lymph node metastasis was associated with disease recurrence, treatment failure, and morbidity. Thus, the PDE5 expression level may in the future help identify patients who are required of aggressive treatment.

## Figures and Tables

**Table 1:** Clinicopathological features of 4 thyroid papillary carcinoma cases.

	Age	Tumor size	Lymph node status	Distant metastasis status
Case 1	30	2 cm	Positive	Negative
Case 2	5	4 cm	Positive	Negative
Case 3	43	1.5 cm	Negative	Negative
Case 4	54	Multiple	Positive	Negative

**Table 2:** Correlation between PDE5 expression and clinicopathological features.

Clinicopathological parameters		Total	The level of PDE5 expression		*P* value
			High (%)	Low (%)	
Gender	Male	36	19 (36.5)	17 (33.3)	0.733
	Female	67	33 (63.5)	34 (66.7)	
Age (years)	<45	67	39 (75.0)	28 (54.9)	**0.032**
	≥45	36	13 (25.0)	23 (45.1)	
Histological type	PTC	100	51 (98.1)	49 (96.1)	0.618
	FTC	3	1 (1.9)	2 (3.9)	
Focus	Single	58	30 (57.7)	28 (54.9)	0.774
	Multiple	43	21 (42.3)	22 (45.1)	
Invasion of capsule or ETE	No	89	45 (86.5)	44 (86.3)	0.969
	Yes	14	7 (13.5)	7 (13.7)	
Distant metastasis	M0	84	47 (90.4)	37 (72.5)	**0.020**
	M1	19	5 (9.6)	14 (27.5)	
LN involvement	N0	46	16 (30.8)	30 (58.8)	**0.004**
	N1	57	36 (69.2)	21 (41.2)	OR = 2.79

ETE: extracapsular extension; PTC: papillary thyroid cancer; FTC: follicular thyroid cancer.

**Table 3:** PDE5 expression and lymph node involvement in patients aged ≥45 years.

LN involvement	The level of PDE5 expression		*P* value
	High (%)	Low (%)	
N0	1 (7.7)	13 (56.5)	**0.005**
N1	12 (92.3)	10 (43.5)	OR = 15.60
